# P-204. Chagas Disease Testing Across Joint Base San Antonio

**DOI:** 10.1093/ofid/ofaf695.426

**Published:** 2026-01-11

**Authors:** Oscar Gallardo-Huizar, Joseph Marcus

**Affiliations:** Brooke Army Medical Center, San Antonio, TX; Brooke Army Medical Center, San Antonio, TX

## Abstract

**Background:**

At least 6 million people in the world are estimated to be infected with Trypanosoma cruzi, including 300, 000 people in the United States alone. These infections can be acquired by close contact with triatome feces, ingestion of food that is contaminated, and blood transfusions. The U.S military has unique factors for increased risk of Chagas Disease including recruitment of service members born in hyperendemic countries, deployments to endemic areas, and bases located where autochthonous transmission has been documented. Previous screening studies within the U.S military population have demonstrated almost no cases of Chagas disease, which may underestimate the true incidence of Chagas disease in a military healthcare system. This study evaluates the current testing practices for Chagas disease at a large base in South Texas.

**Figure:**
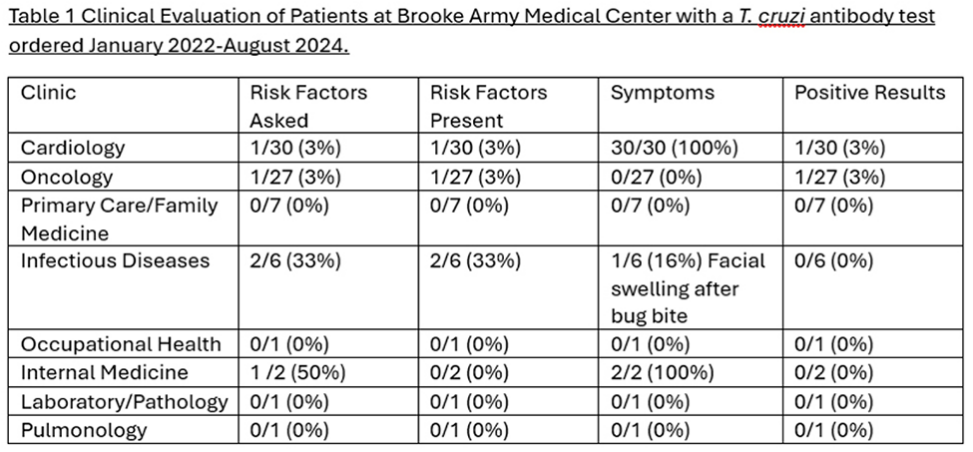
Clinical Evaluation of Patients at Brooke Army Medical Center with a T. cruzi antibody test ordered January 2022-August 2024

**Figure:**
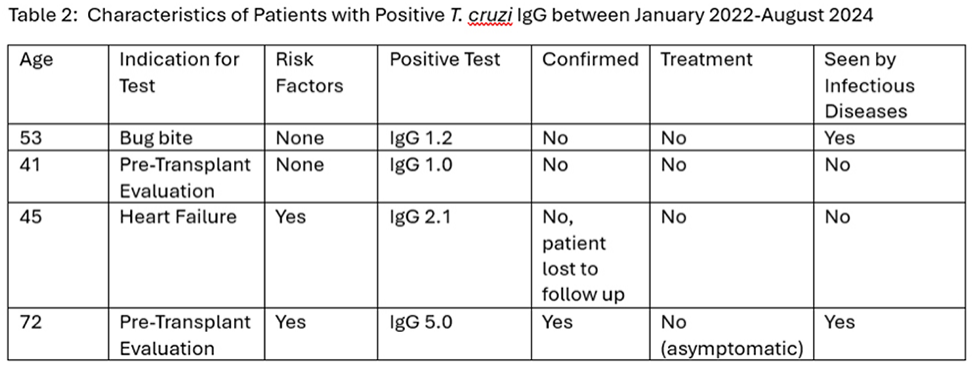
Characteristics of Patients with Positive T. cruzi IgG between January 2022-August 2024

**Methods:**

The electronic health record was queried to identify Chagas tests performed at Joint Base San Antonio between January 2022 – August 2024. For those patients tested, charts were reviewed to document patient factors as well as clinical factors associated with testing. Screening samples from blood donation were not in the electronic medical record and could not be reviewed.

**Results:**

During the study period, there were 75 patients tested for Chagas disease, with 9 (12%) performed on active-duty military personnel. Most patients tested were performed male (75%) and received care in the outpatient setting (72%). Testing was primarily done due to concern for Chagas cardiomyopathy (45%) or as a screening test before bone marrow transplantation (38%). Risk factors for Chagas Disease assessment were only documented for 27 (36%) patients with a test and varied by the specialty ordering the test (Table 1). Four (3%) of patients had an initial positive test (Table 2), but only one (25%), who was tested before bone marrow transplant, had confirmed infection.

**Conclusion:**

This study demonstrates that current testing practices do not target patients with a high pre-test probability of Chagas disease. Consequently, prevalence predictions of Chagas disease based on positive laboratory testing in the military healthcare systems may underestimate the true burden of disease.

**Disclosures:**

All Authors: No reported disclosures

